# Rethinking vulnerability and humanitarian assistance in the pastoral drylands: insights from northern Kenya and southern Ethiopia

**DOI:** 10.1111/disa.70033

**Published:** 2025-12-22

**Authors:** Rahma Hassan, Samuel Derbyshire, Elizabeth Stites, Ian Scoones

**Affiliations:** ^1^ Centre for Research and Development in Drylands Kenya; ^2^ Feinstein International Center Tufts University United States; ^3^ International Livestock Research Institute Kenya; ^4^ Institute of Development Studies University of Sussex United Kingdom

**Keywords:** complex crises, humanitarian assistance, moral economy, pastoralism, relationality, vulnerability

## Abstract

Drought‐related emergency assistance in the drylands is shaped by understandings of vulnerability that are often not commensurate with the socioeconomic dynamics that structure everyday life in pastoralist contexts. Forms of humanitarian assessment and targeting undertaken before the implementation of assistance programmes tend to be oriented towards vulnerability measurements and assessment criteria that focus on individuals or households. These approaches often fail to account for existing local systems of sharing, redistribution, and resource pooling. Recent research into locally‐led social protection, resilience, and livelihood change in the pastoral drylands highlights how pastoralists respond to crises through collective and networked practices, which take on diverse forms but are founded on a common understanding of vulnerability. Differences in how vulnerability is both understood and responded to mean that aid organisations and local communities often do not see eye to eye, which results in mistrust and inefficiencies. This article draws on research undertaken among pastoralist communities in the cross‐border area of northern Kenya and southern Ethiopia to explore local conceptions of vulnerability. In drawing out implications, it asks whether humanitarian agencies might be able to move towards an alternative approach grounded in the more relational, networked understandings of vulnerability that shape life in the drylands.

**Practitioner points**

- **In the drylands, vulnerability is experienced and understood collectively, not just individually**. Aid efforts that assess need and target assistance at the household or individual level are prone to misreading the nature of risk in pastoralist societies, which tends to be shared and managed collectively. One outcome of this is that assistance programmes overlook the networked, relational ways in which pastoralist communities manage crises.
- **Aid that prioritises control often clashes with how pastoralists manage risk**. Efforts to institute control and upward accountability can misread, or fail to engage meaningfully with, the local decision‐making processes and social logics that govern crisis management in pastoralist settings. What aid agencies may interpret as ‘misuse’ of assistance is often, in practice, a socially embedded form of redistribution central to resilience in unpredictable contexts.
- **Context matters more, not less, as aid budgets shrink**. Cost‐cutting pressures may make centralised and standardised approaches to assistance seem more attractive, but these approaches often come at the expense of local relevance, flexibility, and trust. In pastoralist settings, future effectiveness will depend not on tighter control, but on deeper engagement with the local context and community‐led systems.

## 1 INTRODUCTION

Pastoral areas and populations in dryland regions of Africa are frequently cast as vulnerable. Recurrent droughts, exacerbated by climate change, combined with inter‐group conflict and changing land‐use (restricting mobility patterns), have challenged pastoral production systems. Compounded and protracted shocks have resulted in numerous humanitarian crises, triggering national and international interventions to save lives. The *Kenya Drought Response Plan* of 2023, for example, estimated that more than 6.3 million people required humanitarian assistance, with millions of dollars of humanitarian aid channelled to the northern counties, where pastoralism dominates (Government of Kenya, 2023). Much of this was short‐term drought assistance in the form of food aid, emergency nutritional support, cash transfers, and recovery activities in the shape of ‘resilience building’ projects aimed at transforming livelihoods. Then, and now, many humanitarian stakeholders used vulnerability assessments to identify specific individuals and households in need, with assistance thus being targeted directly at these recipients.

These assessments rely on approaches that underlie these types of assistance, which are often modelled on approaches developed elsewhere, particularly in more intensive agricultural areas, where individualised forms of household livelihood based in sedentary contexts are common (Lind, Okenwa, and Scoones, 2020). By contrast, pastoralism is a dynamic livestock‐based production system that relies on the collective and mobile management of rangelands, livestock, and livelihood resources (Niamir‐Fuller, 1998; Maru, 2020; Behnke, 2021; Scoones, 2023a). This collective management system emerges from an equally collective social system, in which sharing and distributing assets across the collective whole are essential for survival in a highly uncertain and variable setting. The critical support that the pastoral system provides humans through these ‘moral economy’ networks of sharing and solidarity has been highlighted by multiple authors (Nori and Davies, 2007; Mohamed, 2023; Scoones, 2023b). However, myriad and overlapping shocks can overwhelm these informal social safety nets, particularly when such shocks extend across multiple seasons or years, such as a multi‐year drought. When this happens, external intervention is often necessary.

Nevertheless, humanitarian interventions frequently harbour an unsound understanding of the local context, with assessments often reflecting a presumed fundamental vulnerability of the overarching system—as recently pointed out by Young et al. (2023). Researchers have made efforts to change this narrative of systemic weakness (Scoones, 2021, 2023a), and the call for a rethinking of humanitarian action to reflect local realities is not new (Robillard, Atim, and Maxwell, 2021; Fitzpatrick, 2024), including work that advocates for new forms of localisation in humanitarian settings (Maxwell et al., 2021).

Yet, research on alternative approaches remains scarce, and few studies include an analysis of the actions of pastoralists themselves during crises. In seeking to fill this gap, this article presents a new case study from the Kenya–Ethiopia border, providing an examination of different understandings (and misunderstandings) of vulnerability, targeting, and collective sharing/redistribution and calling for new forms of external engagement in these settings at a particularly critical time for international aid. We begin by exploring how vulnerability is framed and understood in the world of international development today, and how such framings have been challenged and have changed over time amidst prominent debates and theories. After outlining some of the critical, unexplored implications that emerge from recent research on pastoralism, uncertainty, and relationality, we focus on the specific ways in which vulnerability is targeted by those delivering aid in the study area, and how local communities understand and negotiate these targeting dynamics. Next, we investigate how various forms of aid and assistance come to be enfolded within patterns of locally‐led crisis management based on solidarity and sharing, and how these patterns are transforming over time in relation to wider socioeconomic and ecological changes. We conclude by drawing out wider implications regarding the future of humanitarian assistance in the drylands for pastoralists.

## 2 METHODS

The data analysed here are from a qualitative research study undertaken across multiple sites along the cross‐border area of Marsabit in northern Kenya and the Borena zone of southern Ethiopia (specifically Dambi and Girincho). They were collected in five waves over nine months in 2024, using focus‐group discussions (FGDs), individual open‐ended interviews, and participatory network mapping exercises. Specific research sites were selected to ensure the inclusion of an array of different livelihood dynamics spanning both mobile pastoral communities and pastoralists ‘in transition’, including market exchanges and access or a lack of access to social amenities like roads.

**FIGURE 1:**
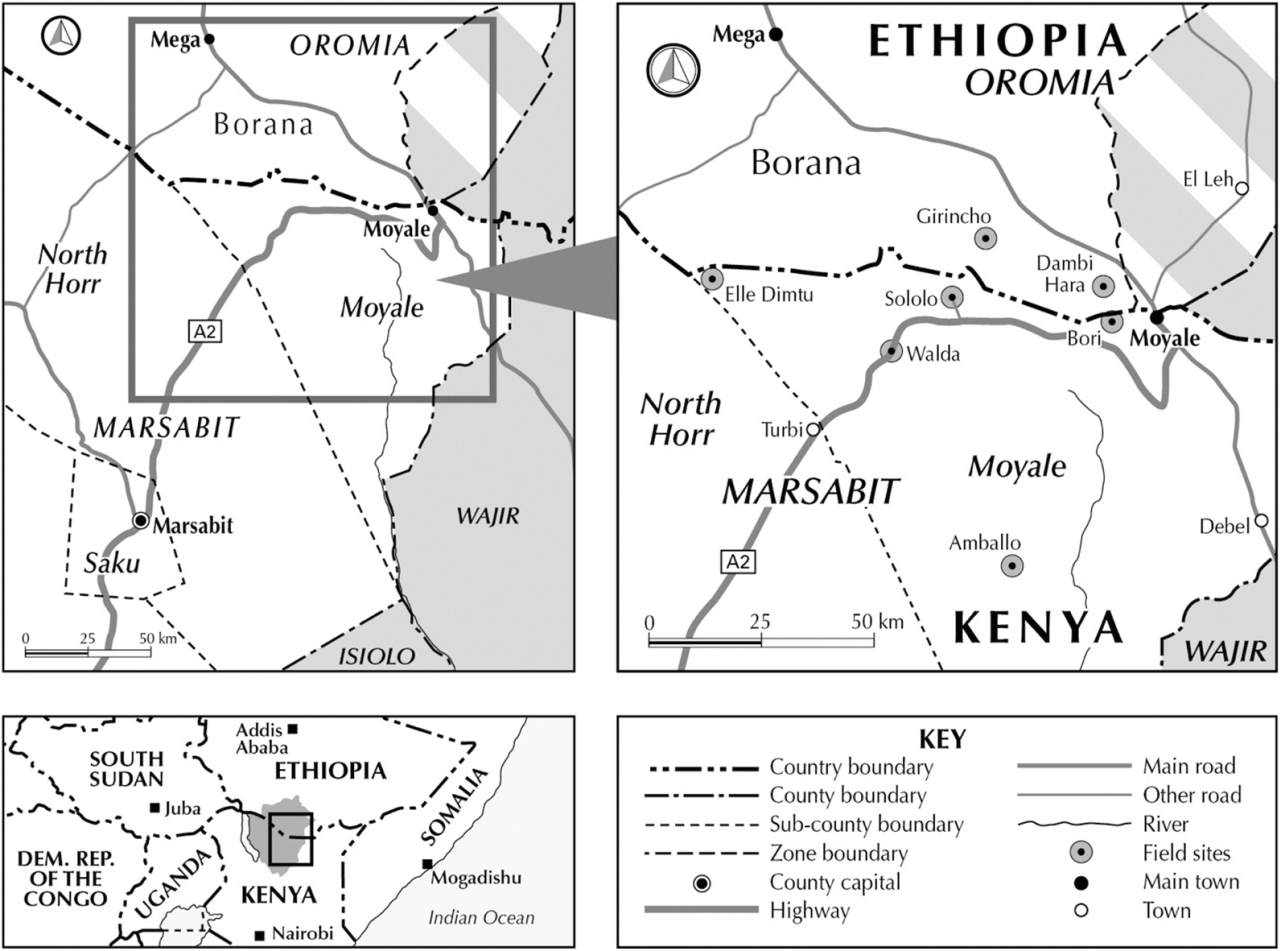
Map showing the location of the fieldwork. **Source:** authors.

Study participants included male and female herders, youth, and local community elders and leaders, as well as local and international humanitarian actors and state officials working on humanitarian and resilience building projects. The topics that were pursued included communities' experiences of shocks and crises, specific tools and knowledge applied via preventative and mitigating strategies, external humanitarian support, and local experiences of assistance, including locally‐led support and community involvement during crises like drought. The participatory tools applied included network mapping, which engendered an understanding of the diverse resources and actors locally and externally, and ranking exercises, which enabled an exploration of the different roles of humanitarian support in the community. Research teams mostly comprised local researchers from the cross‐border area who understand the local language and the different pastoral contexts under review. Data were recorded and later translated and transcribed into English. Analysis of the data was undertaken collaboratively with the teams drawing on daily debriefs in the field and an iterative process of analysis that involved the field research teams and senior researchers. Figure 1 shows the study sites in northern Kenya and southern Ethiopia.

## 3 FRAMING VULNERABILITY

How is vulnerability understood in the development world? And moreover, how do these understandings influence the design, targeting, and monitoring of humanitarian assistance, as implemented by different stakeholders? In practice, such fundamental questions are rarely considered, despite both the critical nature of humanitarian assistance and the fact that, more broadly, wide‐ranging academic discussions have taken shape on both the definition and framing of vulnerability (see, for example, Wisner, 2003; Adger, 2006).

Since its rise to prominence in food security analyses in the 1980s, following the influential work of Amartya Sen (1982), the concept of vulnerability has been highly fraught, with critiques coming from multiple angles in the world of disaster research, and many of them highlighting a prevalent, disproportionate focus on individuals and households at the expense of wider system dynamics. Suggestions for new approaches, many outlined in articles published in this journal, have included a focus on the development of businesses (Zhang, Rezaee, and Zhu, 2010) and more spatially attentive indicators, such as in the case of Ethiopia's Chronic Vulnerability Index (Burg, 2008). Others have advocated for metrics and frameworks that are comparable and replicable across different settings (Alwang, Siegel, and Jorgensen, 2001; Joseph, 2013), or an approach to vulnerability that is oriented towards food systems in their entirety, and uses historical famine dynamics to understand recurring trends (Fraser, 2006). A particularly significant point of criticism of approaches to vulnerability in pastoralist contexts, which, as we have set out, are characterised by high levels of mobility, has been the rigid centrality of government administrative boundaries in various mapping and targeting exercises and associated (although often implicit) assumptions of fixed and permanent residence (Stites, Howe, and Fitzpatrick, 2024).

More standardised approaches to understanding vulnerability have also met with difficult questions concerning how they might effectively cater to diverse and contextually‐specific forms of exclusion and inequality, aims that were, after all, initially central to the concept's early growth and proliferation following work that foregrounded the socioeconomic and political nature of famine (cf. Sen, 1982; de Waal, 2005; Cornwall and Eade, 2010; Bankoff and Hilhorst, 2022). More recent explorations of vulnerability in both thought and practice have thus sought explicitly to centre people alongside their ecosystems, and to understand how capabilities to cope with shocks and crises take shape amidst complex and contingent social processes (Lautze and Raven‐Roberts, 2006). Moreover, Young et al. (2023) have recently pointed to a prevalent opposing focus (in the humanitarian world, and evident in a number of assessments undertaken in pastoral areas) on shocks themselves, and their impact on poor nutrition, food security, and livelihood outcomes. This partial, unnuanced understanding of the core issues at stake results, they argue, in an unhelpful ‘disaster narrative’ that construes pastoralists as stuck in a perpetual state of crisis and vulnerability.

Perhaps most importantly for this article, an enduring vein of criticism vis‐à‐vis vulnerability‐based targeting in the humanitarian world across multiple case studies and reports has been a sense of misalignment between how it approaches problems and what such problems look like from the perspectives of those deemed to be vulnerable. In pastoralist areas, where resources (including land) are, for the most part, communal, and important decisions shaping prosperity, peace, and resilience tend to be vested in the collective, the consequence of individualistic approaches—forms of targeting that foreground individuals, or individual households rather than wider systemic issues or processes—is particularly problematic. Following an initial shift in vulnerability assessment methodologies in the early 2010s towards approaches that focus on resilience and disaster risk reduction, Levine, Barbelet, and Moallin (2025, p. 10) note that humanitarian organisations seem, in more recent years, to have developed different approaches, which ‘use vulnerability as a proxy for needs’ and are oriented much more towards counting the number of vulnerability categories into which particular households fall (see, for example, WFP, UNHCR, and REACH, 2020; Voluntas Policy Advisory, 2021; WFP, 2023). These are the kinds of approaches that often underpin preventive ‘anticipatory action’ protocols for pastoral areas, which seek to respond to risks and vulnerabilities that have been defined using individual‐ and household‐level assessments.

The crucial missing factor in these types of assessment is the socioeconomic networks and support systems that shape life, risk, and resilience for pastoralists. Pastoralist livelihood systems are adaptive and resilient not because they are vast assemblages of individual households, but rather because they are accumulations of relationships that grow around and between resources, landscapes, humans, and animals (Swift, Nori, and Krätli, 2016; Robinson et al., 2017; Derbyshire, 2020). In other words, mobile pastoralism is anchored in systems of mutual support and solidarity that operate within and across communities. When we understand pastoral systems not as a group of separate individuals and disparate households but as a collective network, the nature of vulnerability and the appropriate forms and targeting of emergency response look very different. Pastoral systems function through moral economies, modes of solidarity, the dynamics of sharing and redistribution, and collective arrangements that shape access to resources. Vulnerabilities are seen as collective; that is, shared across people, and are responded to in ways that likewise focus on solidarity, moral economy, sharing, and redistribution (Mohamed, 2023). Indeed, Konaka and Little (2021) have highlighted the need to take a ‘relational approach’ in order to rethink resilience, and Mosse (2013) has done the same in advocating for a rethinking of poverty. Importantly, a relational approach emphasises the complex and changing webs of knowledge and skills that arise through pastoralist livelihoods, calling into question assumptions about any kind of inherent vulnerability (Semplici et al., 2024).

Nevertheless, advocating for a move away from assumptions about the *inherent* vulnerability of pastoralism, as we do in this paper, does not mean imagining that there is no vulnerability within pastoral settings or systems. Variability in the environments that pastoralists occupy (stemming primarily from unpredictable rainfall patterns), along with climate change, market shifts, and political uncertainty, are common features of dryland livelihoods. Pastoralists live with this uncertainty, and make the most of it (Krätli and Schareika, 2010), but major shocks and crises have increased in regularity and severity over recent decades, including conflict, seriously disrupting livelihoods. An interrelated combination of droughts, diseases, and conflicts, coupled with rapid changes in the market, have severely restricted pastoralist's abilities to respond effectively in multiple contexts (Scoones, 2024). In northern Kenya, pastoralist communities have not only faced these rising challenges amidst the legacies of pronounced historic marginalisation, but have also done so alongside a slew of other contemporary risks arising from large‐scale conservation and energy projects targeting resources vital to pastoralism (Lind, Okenwa, and Scoones, 2020; Wachira et al., 2024; Lind and Rogei, 2025). In many places, such schemes have worked to undermine mobility, access to water and pasture, and adaptive capacity (Schilling and Werland, 2023).

Similar trends have been reported in southern Ethiopia, where large‐scale dam and irrigation projects have displaced entire pastoralist populations, dislocating livelihoods and economic relationships (Fratkin, 2014). More widely, Tofu et al. (2025) have underlined the critical impact of climate change on food, land, and health among pastoralists in the Borana region of southern Ethiopia. Overlapping disasters spanning the political, environmental, and economic spheres have served to undermine the social support systems that underpin exchange and sharing (Caravani et al., 2022). A relational approach to vulnerability, however, recognises that collective forms of crisis management oriented towards social networks, kinship, and resource sharing remain—even when temporarily overwhelmed—the most effective means of managing complex challenges at the local level in the long term (Konaka, 2023; Hassan, Stites, and Howe, 2024). In this sense, vulnerability, like ‘resilience’, might be seen less as synchronic, individualised risk, and more as longer‐term processes and social transformations (Scoones, 2023b).

Having said this, it is important to recognise that ‘resilience’ is not without substantive criticisms, chief among which is arguably its tendency (particularly in the realm of international development) to articulate ‘neoliberal’ ideas around self‐reliance and competition (see, for example, Joseph, 2013). In the context of pastoralism—a livelihood still often neglected or undermined by wider economic and political systems—a perennial risk remains in focusing on locally‐led self‐reliance at the expense of an appreciation of the wider systemic failures that engender vulnerability in the first place. In this regard, both resilience and vulnerability are sides of a continuum that, in its entirety, carries a danger of re‐establishing exclusionary, restrictive assumptions about livelihoods, modernity, and development. Our aim in this paper is not to romanticise pastoralist resilience, nor indeed to distract from wider policy‐oriented questions regarding a more just, inclusive future for pastoralists in Ethiopia and Kenya. Rather, while we recognise that most of the issues pastoralists face are either caused or exacerbated by failures in wider governance systems, we also highlight improvements that might be made in approaches to assistance and support through closer articulation of how resilience is achieved at the local level (that is, by envisaging the adaptive capacity of pastoralists as a dynamic that should be drawn on, not undermined). The point here is not to shift onus or responsibility from state agency or non‐governmental organisation (NGO) to community, but instead for wider systems to do better at working in more locally relevant ways.

## 4 TARGETING VULNERABILITY

In our study area and elsewhere, vulnerability is targeted using tools and instruments that focus on gaps, needs, or deficiencies at an individual and household level. The formulation and delivery of assistance likewise follows the same model. Food and cash transfer programmes are oriented either towards household units with particular characteristics (such as female‐headed households, child‐headed households, and internally displaced households) or towards individuals within them who fit certain criteria (such as households with disabled members, elderly members, and individuals with a high dependency ratio). Importantly, both kinds of approach tend to deploy a fairly simple definition of the ‘household’: as people living under one roof and sharing meals (Davies, 2011; Randall and Coast, 2016). This fundamental assumption that fixed‐place households based on nuclear families is a straightforwardly appropriate locus of targeting and support raises serious issues in pastoral settings, where notions of family, obligation, and responsibility tend to reach far beyond individual households. In fact, nuclear family groups rarely act alone, but rather by means of their positions within much broader social contexts and networks, including both clan‐based kinship affiliations and other social and economic relationships (Derbyshire et al., 2021). Being vulnerable in such a setting has less to do with an individual's more immediate circumstances and more to do with their long‐term relationship to the collective. Put another way, a displaced or female‐headed household is not necessarily going to be any more vulnerable than any other kind of household if situated within a community and wider network comprising a strong social safety net based on solidarity and resource sharing.

This was a theme we investigated in detail across all of our field sites. Indeed, during an interview in which it was discussed in August 2024, a humanitarian worker in Marsabit town in northern Kenya explained the process through which lists assembled in relation to externally defined vulnerabilities are adjusted via local committees:

*Once we have received a list of people to be targeted for assistance from the data collected, we rely on the sublocation validation committees who are trusted members of the community selected to examine the list through [a] public process; they include religious leaders, elders, youth, person[s] with disability and other individuals* (KII (key informant interview), Male, Humanitarian Worker, Marsabit, 29 August 2024).

Relatedly, participants in a FGD in the village of Bori, Marsabit, commented on the revisions to beneficiary lists that were likely to take place at the local level, noting how they ‘remove and replace people; we remove a non‐deserving person to put a deserving person. An example of a non‐deserving person is a salaried person who is included in the list’ (FGD, Male, Community Member, Bori, 11 September 2024). A female participant in Ambalo, Marsabit, explained, however, that sometimes local adjustments to such lists were rejected by actors working for aid distributors owing to differing notions of vulnerability. For example, her community had decided that people paying the school fees of secondary or tertiary students should be prioritised for cash transfers, but the donor organisation in question responded that such people were ‘not vulnerable’ and must be excluded. She underlined how ‘the community in this case agrees to follow these rules and present the names as required but we know that we have to look at the list again once we receive this support’ (KII, Female, Community Elder, Ambalo, 5 May 2024). In this instance, the community removed these names from the list in order to receive the aid, but then redistributed the remittances internally once the cash had arrived. Here, as in many other contexts explored in our research, we observed collective support for and prioritisation of children's educational advancement—a means of ameliorating vulnerability in the long term, which nevertheless clashes with the much shorter‐term vision of many external organisations.

Such a flexible reallocation of resources towards collective priorities, rather than individual needs, contrasts not only with the fundamental ideas and assumptions about vulnerability that underpin emergency assistance, but also with the mechanics of its delivery, which are increasingly oriented towards improved control and specificity. In many of our study areas, biometric technology was used to generate lists of recipients, and subsequently to link individuals with the names written on these recipient lists. Many respondents from pastoralist communities emphasised the restrictive nature of this process, and its inability to account for large proportions of the population in less accessible areas or with limited telephone connectivity. In many respects, framing vulnerability in individualised and instrumental terms, as is nowadays so often the case, humanitarian agencies have perhaps inadvertently given rise to a form of ‘governmentality’, which shapes not only movements and choices (often negatively, and particularly by compelling communities to orient themselves towards the processes and mechanisms behind the generation of recipient lists) but also threatens to undermine the dynamics of mutual support that allow people to manage complex crises. In the next section, we build on this idea by investigating these dynamics in more detail, and examining what they reveal about pastoralists' understandings of vulnerability.

## 5 THE LOCUS AND DYNAMICS OF CRISIS MANAGEMENT IN PASTORAL CONTEXTS

While it is important to avoid too monolithic a view of what pastoralism is and how it functions across different sociocultural and economic environments, it is clear that certain characteristics are shared very widely, including, as we have outlined, moral economy practices oriented around customary institutions and kinship structures, which serve as avenues for the redistribution of resources, including livestock (Mohamed, 2023). Such practices are critical to the collective navigation of uncertainty and, importantly, to the management of complex unfolding crises (Scoones, 2021). Their dynamic, flexible nature, which lends itself to the support of improvisation and adaptive decision‐making, also raises significant questions about the modalities shaping assistance programmes (Derbyshire et al., 2024).

Underpinning pastoralist practices in Borana communities is a strong sense of solidarity, which was perhaps best articulated to us by a participant in Sololo, Marsabit, with the succinct saying that ‘the Borana can never be separated’ (KII, Male, Community Elder and Herder, Sololo, 2 June 2024). Among the implications of this saying is a strong sense of cohesion and mutual obligation spanning different landscapes. This view was similarly voiced by a female research participant in Ethiopia, who specifically outlined the centrality of obligation within her community during times of crisis. While recalling the drought of 2021, which caused widespread suffering and crisis levels of hunger throughout the Greater Horn of Africa (Farr et al., 2022), she underscored that ‘when everything was gone and we lost all our livestock, the people who came to our rescue were our fellow Borana who are our neighbours in Kenya and across the globe; even those who had nothing left contributed as it is both a requirement and commitment for us to help each other, especially in crises’ (FGD, Female, Community Member, Dambi, 9 December 2024). It is important to emphasise the geographic expansiveness of the networks invoked here and in many other instances throughout our research. Moral economy practices, and the wider social safety nets to which they connect, tend to cut across diverse social and economic contexts, spanning vast diaspora networks (see also Hughes, 2006; Lesorogol, 2008; Mohamed and Derbyshire, 2024). At the more local level, they include livestock gifting, food, and milk sharing and other rotational systems such as women's savings groups, which are based on trust and social norms (Galvin, 2008; Nori, 2010). The redistribution of cash or food given as emergency aid takes place as part of this much wider, multi‐scalar, and cross‐generational world of morally obligated resource sharing.

This is a world that is, in turn, governed by customary rules and institutions, which likewise cut across boundaries, and are particularly effective when in dire need (Ali and Hobson, 2009). The *Gadaa* system among the Borana of Kenya and Ethiopia, for example, plays a critical role in guiding sharing practices and nurturing rules of support, which are crucial during both conflict and extreme drought—for a more detailed analysis, see Oba (2001). This system of strong collective social relations provides practical solutions in times of crisis (Pollini and Galaty, 2021; Tsegay and Kenton, 2023).

Alongside various forms of sharing, strategic mobility is also central to how pastoralists manage shocks and crises. Herders move their animals both in anticipation of and in response to shocks (Hassan, Stites, and Howe, 2024). In many instances, different ethnic groups traverse shared rangelands, relying on flexible, continually negotiated communal access to pasture and water to navigate periods of difficulty (Scoones, 2021; Jeppesen and Hassan, 2022). Nevertheless, and despite its centrality, Krätli and Swift (2014) have highlighted a tendency for mobility often to be downplayed by respondents during humanitarian surveys (and thus remain uncaptured), which frequently disallow registration of any kind in areas that are not the respondents' ‘usual residence’. This is another dimension of the widespread implicit orientation towards permanent residency and fixed delivery locations for targeted beneficiaries discussed earlier. Alongside the exclusion of those on the move with livestock, which is unfortunate in its own right, this prominent orientation also plays a significant role in processes of sedentarisation around known humanitarian access points (see also Derbyshire et al., 2021). Many scholars have highlighted the diverse negative outcomes of such compelled forms of sedentarisation (Fratkin, 2001; Stites, 2021; Hassan, Kanyinga, and Nathan, 2023).

Like bonds of obligation and mutual support, patterns of mobility regularly cross international borders. Indeed, this dynamic was a key feature of local response to shocks and crises in our study area. Cross‐border collaboration was discussed at length by many of our respondents, including a group of male herders in Elledimtu, northern Kenya, who emphasised the vital role of shared seasonal access to key resources. One said: ‘We always have to cross over to Ethiopia to water our livestock. Even if we have enough pasture here most of the time, we have no water, and we have long shared and accessed their boreholes and watering points since we came here many years ago’ (FGD, Male, Herder, 22 August 2024). In southern Ethiopia, herders described similar movements into Kenya in search of pasture: ‘When the seasons get tough and the rains fail, we all go into the Golbo grazing region in Kenya; if we have to move further south into Kenya we are always welcome’ (FGD, Male, Herder, Dambi, 10 December 2024). These cross‐border dynamics are extremely difficult to account for in formal relief programmes, which, whether run by national governments or other agencies and organisations, almost always fall within national borders and administrative zones. Indeed, a staff member of a humanitarian organisation we interviewed in Marsabit town explained: ‘We know our borderlines and the coordinates, and during the registration and targeting exercises we have GPS [Global Positioning System] gadgets. Even right now I know where my programme officers are, I can locate them from here. So, we cannot aid those who are not from this area’ (KII, Marsabit, 29 August 2024).

## 6 SHARING AMIDST CRISES

Sharing livestock takes many different forms, including permanent transfers, loans of a milking animal, or loans of a pregnant animal and the gift of an offspring (Mohamed, 2023). Importantly, local systems for the sharing or redistribution of livestock require forms of targeting that look very different to the approaches common in formal aid programmes. Prior to livestock distribution, assessments follow collective decision‐making, with responses usually entailing some kind of pooling of resources. For example, when clan members call for support after a drought, the different requests are weighed and a decision is made on which groups will be prioritised according to need (Dahl, 1979). Among Borana groups, these rules align with ‘*buusa gonofa*’, an institution for pooling resources (especially livestock) through different clans for redistribution (Tache, 2008; Cormack, 2016), or with ‘*harambee*’ (a Swahili term technically meaning ‘synergy’ but with connotations of ‘pulling together’), a kind of collective exercise through which funds are gathered and directed towards certain activities or to support groups of pastoralists in crisis (Khalif and Oba, 2018). These processes and decisions are largely male‐dominated as they fall into the male‐controlled realm of livestock management. Nevertheless, as we outline below, many other more localised and female‐managed systems of social support and reciprocity exist among neighbours and community members, which operate both in normal times and during crises (Stites, Humphrey, and Krystalli, 2021; Fitzpatrick et al., 2022).

The sharing of milk, for instance, although related to livestock sharing and transfers, is a much more localised, female‐managed, and daily form of social support and exchange in pastoral communities (Nori, 2019). This daily practice assumes reciprocity when possible but does not require a standard exchange. It might be compared to how an urban apartment dweller might borrow an egg from a neighbour upon realising they are short for a recipe. In this situation asking to ‘borrow’ an egg would not usually mean bringing a replacement egg to the ‘lender’ the next day. The exchange is, rather, based on the implicit promise of reciprocity: that is, we ask each other for assistance with small commodities as needed. Significantly, the domain in which milk is shared has come to encompass the sharing of many other foodstuffs, as diet preferences have shifted to include more purchased cereals and grains (Stites, 2021). Women may exchange cups of maize, sorghum, or beans in the same way that they share milk. While the sharing of milk and other foodstuffs can be a daily occurrence, we also see examples of milk and food sharing being expanded in times of crisis. Recent work by Young et al. (2023) in Marsabit and Isiolo Counties describes the sharing of milk not only as a social and ritual exchange, but also as an action directed at households that had lost animals to drought. Milk was shared for consumption purposes and as a product to sell, thereby enabling the recipient to accrue cash for other needs. Importantly, such forms of sharing and solidarity are not always accessible to everyone who might need them. Indeed, communities or individuals might very well be excluded from reciprocal arrangements altogether, and have few or no social networks from which to seek assistance at all (Little, 2021).

As we have outlined, emergency assistance—even when targeted at individuals or households—is received amidst the same systems and expectations. Food aid, as with milk and purchased commodities, is shared among friends, neighbours, and relatives. The targeting of food aid and other forms of external assistance normally assumes a hierarchy of need, with certain households or categories of household receiving external support and others not being deemed eligible. However, in situations such as severe and protracted drought, and in contexts of pronounced collectivity and sharing, such forms of differentiated vulnerability bear little resemblance to lived experience. A female key informant who also owns goats in Ambalo, Marsabit, described the situation during the recent three‐year drought in her area, stressing that ‘everyone is needy when we all have lost livestock. There is no need of listing the vulnerable because even the rich pastoralists become needy’ (KII, Female, Ambalo, 20 May 2024). In a similar vein, men in a FGD in Bori, Marsabit, spoke about the interconnectedness of people's needs, obligations, and commitments. In the words of one: ‘We all have to be part of each other's problems, and we have lived through supporting each other in groups. Sometimes we support those who help us in return, but other times we help strangers in our community when there is an outcry of help. Sharing and supporting each other is not an option but a rule we have set for ourselves as part of our life’ (FGD, Male, Bori, 14 May 2024).

A number of studies have investigated whether cash is shared in the same way as food when provided in humanitarian contexts, with many indicating that it is shared less often, less generously, or less widely as compared to food aid. For example, examining sharing in refugee camps in northern Uganda, Stites, Humphrey, and Krystalli (2021) found that, while cash and food aid were both shared, cash was shared with fewer people and most often with relatives and close friends as opposed to among the circle of neighbours with whom food was commonly distributed. In contrast to these findings, respondents in our study reported the widespread sharing of cash transfers. In northern Kenya, individuals deemed vulnerable following assessments receive cash through the national Hunger Safety Net Programme, as well as through transfers during the drought and recovery period from a number of national and international NGOs. In southern Ethiopia, the long‐running Productive Safety Net Programme (PSNP) provides regular cash support. Respondents in this study across both areas suggested a prevailing sense that those who receive cash transfers treat the resource like any other—that is, something that is subject to the same rules and precedents that shape wider moral economy practices. This is reflected in findings from pastoral areas of Ethiopia where clan‐based and mutual support networks were found to play a critical role in how communities share, distribute, and negotiate assistance (Sabates‐Wheeler, Lind, and Hoddinott, 2013; Lind et al., 2022). More research is required, however, to understand how widespread such dynamics are across pastoralist settings, and how the sharing of cash differs from the sharing of food in other places.

We asked participants to explain in more depth how food or cash assistance received from outside sources is reconfigured to account for locally‐defined needs that diverge from those captured in vulnerability assessments. Answers differed from one community to the next based on context and collective appraisals of what the greatest needs were. For example, a member of a male FGD in Elledimtu, northern Kenya, said that what happens to the aid once it is delivered ‘is purely *our* plan. The local organisations delivering aid would mostly target 30 or 40 households out of over 100 in our area. The selected households are not the only ones who are needy and some of them may have other sources of support. … The community then decides how the assistance received will be redistributed by looking afresh at who is most needy or has other pressing needs’ (FGD, Male, Elledimtu, 21 August 2024). As shown by the comments of another participant in the same FGD, the criteria for redistribution may look similar to external vulnerability criteria: ‘We consider different things at different times, but currently we ask households who have received cash transfers and food to consider those with many young children, those who have no livestock left, those who have not been given any livestock to restock, and those whose children are unemployed’ (FGD, Male, Elledimtu, 21 August 2024). In such instances, community‐based discussions and decision‐making following initial distributions do not necessarily seek to challenge externally‐derived criteria (as was the case with the previous example of prioritisation for those paying school fees), but rather to spread assistance over a greater number of vulnerable households, including some who may not have been captured by external vulnerability targeting assessments.

Respondents also highlighted that the rules of redistribution were flexible, and that the process varied based both on the aid received and prevailing needs. For instance, a male FGD in Girincho, southern Ethiopia, focused on cases when only small amounts of assistance were received. When this aid is not enough to redistribute widely, respondents noted that a broad‐based decision is made for recipients simply to share with those close to them. One participant remarked: ‘Those who receive small amounts of cash … during drought sometimes buy small foodstuffs for their relatives or neighbours, like one kilo[gram] of sugar and flour, instead of sharing the small amount of cash received’ (FGD, Male, Girincho, 28 August 2024). Clearly, the rules of sharing, redistribution, and locally‐led targeting remain flexible, but are nevertheless subject to accepted practices of vulnerability assessment.

External assistance is also shared through self‐help groups such as village savings and loan associations and similar entities. A group of women in southern Ethiopia, for example, described their rotating support group, which saw them share foodstuffs from their households, including items received via relief distributions. Their sharing targeted members of their group, but also reached wider community members who had no food or had not received relief food. Members also made regular cash contributions to the group, which came both from the sale of livestock and from cash transfer programmes. Small cash grants were then distributed to other members on a rotating basis. Members of the group we interviewed described this form of social and financial support as one of the ways they managed to support their families during the last drought.

Women in Ambalo, northern Kenya, described similar savings and credit groups, such as the Ambalo women's group, whose members were almost all recipients of cash transfers from a local humanitarian organisation. Members of this group pooled their funds to cover shifting food security needs, the payment of secondary school fees, and to allow for borrowing when other urgent needs arose. Women in such groups who are not beneficiaries of humanitarian cash transfer programmes are supported using these collective funds. Reputation and social capital allow the group to raise funds in a number of ways to respond rapidly to a shifting terrain of needs, including via local shopkeepers. One woman commented: ‘We collect part of the cash as women groups and sometimes we borrow the total amount from trusted shopkeepers in our locality. Once we receive the cash transfers and contributions from members we refund the shopkeeper … we have this agreement that helps us continue supporting each other’ (FGD, Female, Ambalo, 28 August 2024). Those on a cash transfer list are seen as credit‐worthy for obvious reasons; some shopkeepers will also let others buy food on credit, knowing that funds are coming into the group at the end of the month. In this way, cash transfers are incorporated into local community‐level support systems to act as security for other community members, enabling them to access credit.

Another form of mutual support identified during our study was fodder groups, which were active along the cross‐border escarpment between Kenya and Ethiopia. These groups comprise both men and women and provide a platform for the pooling of resources with a view to growing, harvesting, and storing fodder, which is then used either to feed group members' own herds or to sell for profit during drought periods. Members benefit from an equal sharing of profits from sales. In Marsabit town and Sololo, northern Kenya, participants who were members of different fodder groups reported receiving external humanitarian assistance during drought, including animal feeds and storage for fodder. They often used these inputs to strengthen their groups.

The examples explored here are only a few of the many ways in which the communities in our study area collaborate to navigate volatile conditions, including protracted and complex crises. While we have referred to different understandings of and approaches to vulnerability in fairly binary terms (that is, ‘external’ versus ‘internal’ approaches), it is important to emphasise that such binary terminology (while useful for the sake of clarity) is often unhelpful when it comes to understanding the values, relationships, precedents, and social rules that actually underpin how pastoralists navigate difficult conditions. In reality, any form of support derived from the aid system comes to be immediately enfolded within these currents of life at the point of receipt. In this sense, it tends not to be conceptualised as different in any fundamental sense to any of the other resources that are drawn on through collective action: the matter of its origins is irrelevant.

## 7 THE CHANGING DYNAMICS OF VULNERABILITY AND ASSISTANCE

Over the past few decades, pastoralist communities across multiple regions of Africa have experienced substantial socioeconomic, ecological, and political transformations, often directly impacting the dynamics of mutual assistance and support that are critical during crises. In many settings, including our study area, networks and relationships that previously facilitated the sharing and redistribution of livestock have been weakened owing to reducing stock numbers. The rules of mutual assistance have become harder to apply to herding families that are regularly too poor to reciprocate. Likewise, questions remain regarding the ways in which socially prescribed forms of obligation—where they do endure—may be enfolded within unequal power dynamics and how these, in turn, may be developing in negative ways in relation to wider socioeconomic factors and forces. As noted earlier, it is important to avoid envisaging pastoralism in monolithic, unnuanced terms. It is equally important not to essentialise a moral world that, for many, may feasibly lead to restriction, dissatisfaction, additional burden, or even exclusion as much as it does assistance or support. While such possibilities were not often raised by participants in our research, we are nevertheless cognisant of them. Our argument in this paper is that solidarity and kin‐based sharing practices are important dynamics that already structure local approaches to unpredictable conditions, and that these dynamics should therefore, in a straightforward sense, feature more prominently in considerations of how best to assist and support from the ‘outside’. As we set out in the conclusion, this should not mean developing new, equally generic approaches or metrics that attempt to cater to drylands pastoralism in general. Nor should it mean imagining that such practices as the ones we have described cater to all needs and ambitions. In northern Kenya and southern Ethiopia, these practices are part and parcel of a social context in which terms like crisis and prosperity may come with different connotations, values, and assumptions altogether. Taking this world seriously in development planning and practice means taking individual contexts on their own terms, not construing them in universalising, generalising terms or imagining that they contain simple comprehensively accepted solutions.

Much empirical work on moral economy practices in pastoralist settings has described reconfigurations in both the form and means of locally‐led social support and mutual assistance. Previous kin‐ and community‐based ties of support have, in many instances, shifted as a result of disruptions caused by severe drought, with new alliances being forged beyond livestock and family ties. While still guided by core principles of solidarity and mutual assistance, networks of support activated during drought emergencies to cater to shifting needs have grown beyond the level of clan and stock associates to include collectives working on mutual projects (like savings groups or fodder production) or linkages beyond more immediate community settings that cross borders and reach new markets, resources, and services. Such new approaches, many examples of which we discussed in the preceding section, are on one level closely linked with heightened challenges and difficulties, including those associated with climate change. Yet, we would argue that they are also articulations of a fundamental capacity common to many pastoralist communities for establishing long‐term continuities in overarching orientations towards the world and towards other people amidst the turbulence of more immediate disjuncture and change (cf. Derbyshire, 2020).

In other words, we suggest that there is space for new ways of thinking about pastoralist solidarity and mutual support beyond notions of ‘erosion’ and ‘breakdown’, which emphasise rising trends of exclusion and diminishing access to assistance for the poor (Catley and Iyasu, 2010; Coppock, 2016). While pastoral systems may, in many cases, be in the process of becoming more stratified and indeed may be struggling to manage more severe drought crises, it is also critical to pay attention to how practices that are socially supportive within new contexts, across new networks, and at new scales are emerging to fill the gap of pre‐existing approaches. An important finding from our study is that vulnerability continues to be framed in relational terms, and responses emerge through collective practices based on complex networks of support and redistribution within and across communities. These fundamental features have persisted, with new institutional innovations showing the same relational characteristics. The rules and principles of solidarity, sharing, and care continue to shape everyday life among Borana communities in northern Kenya and southern Ethiopia, as in other pastoral societies (Scoones, 2023b).

Such innovations have, in many contexts, taken shape around the emergence of new urban centres—a critical dynamic in ongoing processes of socioeconomic transformation across the drylands of the Greater Horn of Africa and beyond. Some participants in our study described how they had formed new alliances in urban environments to gain access not only to markets but also to humanitarian assistance at distribution centres through relatives. In Sololo town centre in Marsabit, women sell camel milk along the main highway to travellers and link with their families and networks. Our study participants emphasised that most of this milk is transported there from more rural regions for sale. During drought, urban areas are a common distribution centre for food aid, fodder, and water. Nowadays, those from more rural areas regularly liaise with friends and relations in urban areas for information on the availability of such resources, as well as to share the assistance received by households in these places. The prevailing sense is not necessarily of urban growth as a force that threatens to disrupt previous patterns and practices, but rather as a source of new economic opportunities, by means of rules and principles that are long held and remain vital to the functioning of the pastoral economy.

The enduring prominence and success of networked, relational, and collective responses to vulnerability that draw on diverse resources to respond nimbly to a changing terrain of challenges, as described in this article, harbours important implications for humanitarian activities in multiple pastoralist settings.

## 8 CONCLUSION: THE FUTURE OF HUMANITARIAN ASSISTANCE IN THE DRYLANDS

Fundamentally, our findings underline the value and importance of ‘taking context seriously’ when approaching the question of vulnerability in the pastoral drylands. Comparisons might be drawn with Pain and Levine's (2024) recent discussion of Community Development Councils during post‐conflict reconstruction in Afghanistan, where, despite the investment of vast sums of money, the ambition of fostering democracy and greater state legitimacy were left unrealised by aid programming that utilised a universalising model copied from another country and failed to account for existing institutions and power dynamics at the village level. In the same way, plans implemented in northern Kenya that do not include serious consideration of the distinct socioeconomic dynamics, values, and interests that shape crisis management and response are likely to have less impact.

Writing in this journal more than a quarter of a century ago, and using evidence from several contexts in eastern Africa, Jaspars and Shoham (1999, pp. 360) advocated for a similar focus on contextual specificity, and for less emphasis to be placed on household‐based vulnerability targeting altogether. ‘Complex emergencies are not’, they argued, ‘temporary interruptions in a linear development process, but rather systemic and protracted crises’. The kinds of vulnerability that are important amidst them are not usually determinable via targeting exercises that deploy generic metrics or criteria. The aid sector has transformed substantially since this article was published, and indeed is in the process of particularly seismic transformation at present, and yet household‐based targeting often still prevails in pastoral settings. At a macro level, one reason for this is perhaps a deep‐rooted inclination towards higher levels of control (over data, decisions, and outcomes), which itself arises from an orientation towards risk that envisages a ‘return to conditions of predictability’ as the ideal (Caravani et al., 2022, p. 6). The kinds of upward accountability and rigid bureaucratic processes that go along with such an orientation make it extremely difficult for those providing assistance—whether via a civil service ministry, a government agency or department, or an international organisation—to embrace flexibility, experimentation, or improvisation. In the case of emergency assistance in northern Kenya and southern Ethiopia, programmes seem to have extremely limited capacity for opening up to the locally‐led forms of resource sharing and assistance that may not align with organisational perspectives, but which nevertheless do reflect both a careful and considered understanding of the key vulnerabilities faced, and a rich collective experience of how best to confront them.

Arguing along similar lines, and in relation to recent usage of vulnerability assessments in Somalia and Ukraine, Levine, Barbelet, and Moallin (2025) have advocated for management‐led cultural shifts that may enable organisations to get better at embracing imperfection as opposed to viewing deviations from pre‐set plans as automatic failures. As part of this, difficult, perhaps even uncomfortable, conversations must be had about what is and is not working, and how to embrace uncertainty more fundamentally in disaster response and humanitarian support settings (Scoones, 2024). In the drylands, where pastoralist societies share the resources that are critical to the success of their livelihoods and manage uncertain conditions, including complex crises, through collective actions based on solidarity, organisations seeking to provide assistance must reprioritise. Instead of aiming for more control, or more generic and systematised programming, they must seek genuinely to engage with local perspectives, and find ways of addressing the kinds of (relational) vulnerability that make sense to recipients.

In doing so, it is important to examine more closely the assumptions that shape conceptions of vulnerability at the institutional level, and to ask whether they are really commensurate with those underlying pastoral livelihoods. A key departure point here is perhaps Ellen Gordon‐Bouvier's (2020) recent work on ‘relational vulnerability’, which draws on socio‐legal and critical studies to approach vulnerability in terms of human relational and institutional embeddedness. This work counters common institutional and state perspectives that imagine ideal autonomous liberal subjects who are entirely self‐sufficient, and ‘pathologize vulnerability and dependency as weakness, symbolic of a failure to attain autonomous personhood’ (Gordon‐Bouvier, 2020, p. 4). Vulnerability, argues Gordon‐Bouvier (2020), is not a straightforwardly solvable problem but a common feature of all human lives, whose qualities are fundamentally shaped via relations (cf. Korzenevica et al., 2024). Being resilient, in this sense (and particularly in pastoralist contexts), is not so much about what a person has in their bank account (or indeed their mobile money account), but much more about the characteristics of the relations that exist between the person and his or her wider networks (Semplici et al., 2024).

This is not to say that financial assistance is misplaced. To the contrary, our study highlights the vital role that cash transfer programmes have played in addressing critical issues (many of which relate both to historic marginalisation and livelihood pressure associated with climate change) and helping people weather difficult times through the incorporation of such cash into all kinds of innovative groups, plans, and businesses. Notably, in almost all instances, this has been achieved by drawing cash received by individuals into collective decision‐making processes and converting individual and household lists and priorities used for targeting, which make sense amidst aid sector rationalities, into outcomes that make sense through lived realities. It would be better, and likely more cost effective, if this reallocation of cash was not necessary in the first place. Thus, as aid budgets are reduced and pressure to improve efficiency and cost effectiveness rises, it is important that progress towards more localised, participatory approaches to vulnerability are not abandoned in favour of approaches that—usually only superficially—seem easier to control and predict.

## Data Availability

The data that support the findings of this study are available on request from the corresponding author. The data are not publicly available due to privacy or ethical restrictions.
